# Evaluating the Effect of Total Hip Arthroplasty on Bone Mineral Density in Postmenopausal Women Using an Artificial Intelligence-Assisted Osteoporosis Diagnostic System

**DOI:** 10.7759/cureus.87396

**Published:** 2025-07-06

**Authors:** Hisatoshi Ishikura, Toru Moro, Takeyuki Tanaka, Naoto Kaminaga, Kenichi Kato, Mayu Iiboshi, Sakae Tanaka

**Affiliations:** 1 Department of Orthopedics, The University of Tokyo, Tokyo, JPN; 2 Department of Orthopedic Surgery, The University of Tokyo, Tokyo, JPN

**Keywords:** artificial intelligence, bone mineral density, chest radiograph, osteoarthritis, osteoporosis, total hip arthroplasty

## Abstract

Background and aim: Total hip arthroplasty (THA) alleviates pain and improves walking ability and quality of life in patients with hip osteoarthritis (OA). However, its effect on systemic bone mineral density (BMD) remains unclear. In this study, we investigated the effect of THA on systemic BMD in postmenopausal women with hip OA using an artificial intelligence (AI)-assisted osteoporosis diagnostic support system that estimates BMD solely using posteroanterior chest radiographs.

Methods: This retrospective observational study included postmenopausal Japanese women aged 50-59 years who underwent bilateral THA on separate occasions at our institution between 2007 and 2023. BMD was estimated using our AI-assisted osteoporosis diagnostic system that uses posteroanterior chest radiographs obtained approximately one month before each THA. The rate of change was compared with age-specific BMD reference values for the Japanese population.

Results: A total of 59 patients were included, with a mean age of 54.9 years and an average interval between unilateral THA of 2.1 years. Among those who underwent an initial THA at ages 50-54 years, the estimated BMD decreased by 1.52% and 0.81% annually in the lumbar spine and proximal femur, respectively. For patients who underwent an initial THA at ages 55-59 years, the estimated BMD decreased by 0.00% and 0.80% annually in the lumbar spine and proximal femur, respectively. These rates were lower than the reported rates of annual BMD decline in Japanese women aged 50-54 years (1.77%, lumbar spine; 1.27%, proximal femur) and 55-59 years (1.28%, lumbar spine; 0.88%, proximal femur).

Conclusion: THA has the potential to attenuate age-related BMD decline in postmenopausal women with hip OA.

## Introduction

Hip osteoarthritis (OA) is a leading cause of hip pain, gait disturbance, and impairment in activities of daily living [1]. Total hip arthroplasty (THA) is known to alleviate hip pain, improve walking ability, and improve quality of life and cardiopulmonary function [2,3].

An aging society has led to an annual increase in the number of patients with osteoporosis. Fragility fractures, such as vertebral and proximal femur fractures, not only decrease activities of daily living but also affect life expectancy in these patients [4]. Fractures around implants after THA often require large plates or implant replacements, resulting in extensive treatment. In particular, postmenopausal women experience a substantial decrease in bone mineral density (BMD), highlighting the importance of appropriate nutrition, exercise, and osteoporosis treatments during this period [5]. Regarding the postoperative effects of THA, there have been reports of local bone remodeling around the stem [6]; however, there is still no consensus on its effect on systemic BMD. To address these osteoporosis-related issues, an artificial intelligence (AI)-assisted osteoporosis diagnostic system has been developed to estimate BMD in the lumbar spine and proximal femur using posteroanterior chest radiographs [7,8]. This study aimed to verify the effect of THA on BMD in postmenopausal women using this system and preoperative chest radiographs. Our hypothesis is that THA attenuates age-related BMD decline in postmenopausal women.

## Materials and methods

Patients data

We conducted a retrospective observational study in postmenopausal Japanese women aged 50-59 years who underwent independent bilateral THA at our institution between 2007 and 2023. Menopause was defined as the absence of menstruation for more than one year, and menopausal status was determined based on self-reported information. The exclusion criteria were as follows: patients with less than six months or more than 10 years between bilateral THA, patients with obvious abnormalities on chest radiographs (e.g., pneumonia) or foreign bodies (e.g., pacemakers), and patients using osteoporosis treatment drugs during the observation period. As a result, a total of 59 patients were included in the study, comprising 23 patients aged 50-54 years and 36 patients aged 55-59 years. The mean age was 54.9 years, and the mean interval between the two unilateral THAs was 2.1 years. The patient demographics are summarized in Table 1. Written informed consent was obtained from all study participants. The study protocol was approved by the institutional review boards of our institution (Ethical Committee approval no.: 11953-7).

**Table 1:** Patient demographics. BMD: bone mineral density; THA: total hip arthroplasty

Variable	50-54 years age group	55-59 years age group	P-value
Number of patients	23	36	
Age at the first THA (years)	52.1±1.3	56.7±1.5	<0.001
Height (m)	1.56±0.08	1.57±0.09	0.602
Weight (kg)	57.7±12.2	57.0±11.8	0.145
Body mass index (kg/m^2^)	23.3±3.9	23.5±3.8	0.387
Interval between THAs (years)	1.7±1.1	2.3±2.3	0.2
Pre-THA			
Estimated BMD in lumbar (g/cm^2^)	1.11±0.12	1.02±0.12	0.01
Estimated BMD in femur (g/cm^2^)	0.92±0.08	0.87±0.10	0.04
Post-THA			
Estimated BMD in lumbar (g/cm^2^)	1.08±0.12	1.02±0.12	0.08
Estimated BMD in femur (g/cm^2^)	0.91±0.08	0.86±0.10	0.01

AI-assisted osteoporosis diagnostic system

The authors have conducted the research and development of this AI-assisted osteoporosis diagnostic system using cohort data from approximately 50,000 individuals who underwent health check-ups at hospitals and about 20,000 individuals from the general population [8]. This system performs regression analysis within seconds of uploading a single chest or lumbar spine radiograph and provides estimates of BMD, young adult mean percentage, and T-scores for the lumbar spine and proximal femur. Previous studies have reported highly accurate estimation results using this system [7-9]. Specifically, the correlation coefficients (CCs) between the estimated BMD values calculated from anterior chest radiographs and the actual measured values for the lumbar spine and proximal femur were 0.85 and 0.84, respectively, which were higher than those achieved by other methods, such as dual-energy X-ray absorptiometry (DXA) of the forearm (CC: 0.46-0.62), microdensitometry of the metacarpal bone (CC: 0.66), and quantitative ultrasonography of the calcaneus (CC: 0.29-0.55) [10-14].

Image inspection

Using posteroanterior chest radiographs obtained approximately one month before each unilateral THA, we calculated the estimated BMD from the first and second THA preoperative chest radiographs using the AI osteoporosis diagnostic support system. The BMD calculated from the preoperative chest radiographs for the first THA was considered the pre-THA BMD, whereas the BMD calculated from the preoperative chest radiographs for the second THA was considered the BMD after unilateral THA (post-THA). We compared the rate of change with age-specific BMD reference values for the general Japanese population to assess the effect of THA on BMD [5].

Data analysis

All statistical analyses were performed using IBM SPSS Statistics for Windows, Version 24 (Released 2017; IBM Corp., Armonk, New York, United States). Statistical significance was set at p < 0.05. The student’s t-test was used to compare the differences in the mean values between the two groups.

## Results

Before surgery, the mean estimated BMD values for the lumbar spine and proximal femur were 1.06 g/cm^2^ and 0.89 g/cm^2^, respectively. After THA, with an average follow-up of 2.1 years, the mean estimated BMD values were 1.05 g/cm^2^ and 0.88 g/cm^2^ for the lumbar spine and proximal femur, respectively, showing a slight decrease compared with preoperative values.

**Figure 1:**
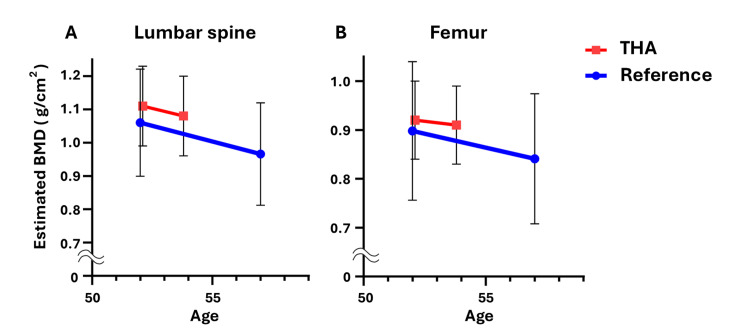
Trend of the estimated BMD in THA patients aged 50-54 years. The trend of mean and standard deviation of the estimated BMD of the lumbar spine (A) and proximal femur (B) in primary THA patients aged 50-54 years (red line), along with the reference values in Japanese women of the same age (blue line). BMD: bone mineral density; THA: total hip arthroplasty

**Figure 2:**
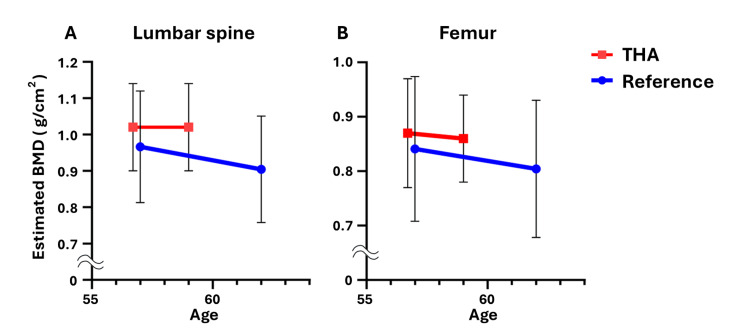
Trend of the estimated BMD in THA patients aged 55-59 years. The trend of mean and standard deviation of the estimated BMD of the lumbar spine (A) and proximal femur (B) in primary THA patients aged 55-59 years (red line), along with the reference values in Japanese women of the same age (blue line). BMD: bone mineral density; THA: total hip arthroplasty

In this study, patients were divided into two groups: those aged 50-54 years and those aged 55-59 years. Each group was compared with the BMD reference values of the same age group in the general population. Among the 23 patients in the 50- to 54-year age group who underwent primary THA, the estimated BMD decreased by 1.52% (lumbar spine) and 0.81% (femur) annually (Figure 1). Among the 36 patients in the 55-59 age group who underwent primary THA, the estimated BMD decreased by 0.00% (lumbar spine) and 0.80% (femur) annually (Figure 2). These rates were lower than the age-specific annual BMD loss rates reported in the literature, which indicated an annual bone density decrease rate of 1.77% (lumbar spine) and 1.27% (femur) per year for Japanese women aged 50-54 years and 1.28% (lumbar spine) and 0.88% (femur) per year for those aged 55-59 years (Figures 1, 2) [5]. The detailed data for each case are presented in Tables 2-3.

**Table 2:** Estimated BMD and YAM values before and after THA in individuals aged 50-54 years. BMD: bone mineral density; YAM: young adult mean; THA: total hip arthroplasty

Age (years)	THA interval (days)	Estimated BMD in lumbar spine				Estimated BMD in femur			
		Before THA		After THA		Before THA		After THA	
		BMD (g/cm^2^)	YAM (%)	BMD (g/cm^2^)	YAM (%)	BMD (g/cm^2^)	YAM (%)	BMD (g/cm^2^)	YAM (%)
50	216	1.13	99	1.05	91	0.91	95	0.91	95
50	762	0.99	86	0.95	82	0.84	88	0.87	90
51	291	1.10	95	1.08	94	0.94	97	0.97	101
51	327	1.18	102	1.05	91	0.84	87	0.83	86
51	974	1.11	96	1.07	93	0.96	100	0.91	95
51	301	1.11	96	1.11	96	0.92	96	0.88	92
51	654	1.18	102	1.26	109	0.99	103	0.99	103
51	435	1.15	100	1.07	93	0.93	96	0.87	91
52	923	1.08	94	1.01	87	0.85	89	0.87	91
52	847	0.96	83	0.93	81	0.90	94	0.82	85
52	287	1.29	112	1.26	109	0.98	102	0.96	100
52	1638	1.10	96	1.07	93	0.99	104	0.95	99
52	840	1.24	108	1.09	95	0.91	95	0.87	90
52	217	0.93	81	0.99	86	0.81	84	0.84	87
52	241	1.06	92	0.98	85	0.90	94	0.90	94
53	420	1.05	91	1.03	89	0.92	96	0.91	95
53	1,561	1.01	88	1.03	90	0.88	91	0.90	94
53	441	1.19	103	1.20	105	0.99	103	1.01	105
54	357	1.19	104	1.20	104	0.99	103	0.97	101
54	1022	0.89	78	0.86	75	0.80	84	0.79	82
54	256	1.28	111	1.23	107	1.02	106	1.00	104
54	623	0.93	80	0.96	83	0.83	86	0.83	87
54	252	1.32	114	1.37	119	1.15	120	1.12	117

**Table 3:** Estimated BMD and YAM values before and after THA in individuals aged 55-59 years. BMD: bone mineral density; YAM: young adult mean; THA: total hip arthroplasty

Age (years)	THA interval (days)	Estimated BMD in lumbar spine				Estimated BMD in femur			
		Before THA		After THA		Before THA		After THA	
		BMD (g/cm^2^)	YAM (%)	BMD (g/cm^2^)	YAM (%)	BMD (g/cm^2^)	YAM (%)	BMD (g/cm^2^)	YAM (%)
55	280	1.15	100	1.25	109	1.00	104	1.00	104
55	469	0.91	79	0.92	80	0.73	76	0.73	76
55	945	1.20	104	1.04	90	1.10	114	1.04	108
55	397	0.91	79	0.92	80	0.87	90	0.84	88
55	376	0.92	80	0.96	83	0.83	86	0.84	88
55	1163	1.13	98	1.15	100	0.98	102	0.93	97
55	427	0.93	81	1.00	87	0.72	75	0.80	83
55	217	0.96	83	0.96	84	0.87	90	0.86	90
55	189	1.19	104	1.22	106	1.01	105	0.97	101
55	358	1.06	92	1.01	88	0.93	96	0.88	92
55	410	1.24	108	1.12	97	0.93	96	0.88	92
56	302	0.96	83	0.93	80	0.82	85	0.80	83
56	2366	1.20	104	1.23	107	1.04	108	0.96	100
56	1491	0.82	71	0.74	64	0.75	78	0.70	72
56	204	0.93	81	1.03	89	0.85	89	0.86	89
56	2976	0.88	76	0.86	75	0.82	85	0.77	80
56	329	0.94	82	0.98	85	0.82	85	0.82	85
56	1701	1.03	90	0.99	86	0.78	81	0.77	80
56	567	0.92	80	1.08	94	0.81	84	0.86	90
56	238	0.95	83	1.00	87	0.87	90	0.90	94
57	2711	1.09	95	1.10	95	1.00	104	0.91	95
57	280	1.00	87	0.92	80	0.88	92	0.83	86
57	212	0.93	81	1.03	89	0.85	89	0.90	94
58	2934	0.85	74	0.80	70	0.75	79	0.68	71
58	721	1.03	89	1.01	88	0.85	88	0.83	87
58	299	0.98	85	0.97	84	0.78	81	0.78	81
58	869	1.13	98	1.17	102	1.00	104	0.99	103
58	2176	0.95	82	1.01	88	0.93	97	0.88	92
58	667	1.05	91	1.06	92	0.85	88	0.84	88
58	259	1.22	106	1.11	96	0.92	95	0.85	88
59	252	1.13	98	1.20	104	0.84	87	0.90	94
59	260	1.04	90	1.01	88	0.87	91	0.90	94
59	728	1.19	103	1.18	103	0.99	103	0.92	96
59	2095	0.84	73	0.82	71	0.71	74	0.71	74
59	381	1.17	101	1.02	88	0.96	99	0.89	93
59	354	0.96	83	0.99	86	0.77	80	0.81	84

## Discussion

Our study highlights the potential for THA to mitigate age-related BMD decline in postmenopausal women. Few studies have investigated the relationship between THA and systemic bone density, potentially because of the inaccuracy of DXA measurements at the same site in patients with hip OA and the impossibility of DXA measurements at the same site in patients with post-THA [15,16]. However, assessing BMD both before and after THA is crucial because poor bone quality negatively affects postoperative outcomes [17]. Monitoring the bone status of patients undergoing THA and prompt intervention for osteoporosis may improve THA outcomes. Meta-analyses have demonstrated the effectiveness of osteoporosis medications in preventing bone loss around implants [18].

The AI-assisted osteoporosis diagnostic system, capable of estimating BMD using chest radiographs commonly obtained during routine health checkups, offers advantages in terms of medical costs and serves as a useful screening tool for osteoporosis. Its key advantage lies in its ability to accurately estimate BMD using past chest radiographs, regardless of the lumbar spine or hip joint condition. Using this system, we were able to track BMD changes in patients over time, from pre- to post-THA.

Factors that may have contributed to the slow decrease in BMD in postmenopausal patients undergoing THA observed in our study include increased physical activity owing to pain relief and rehabilitation during the perioperative period, which may have improved awareness of physical health. Numerous studies have indicated the beneficial effects of exercise on BMD [19].

Our study has several limitations. First, we could not eliminate errors inherent in the AI-assisted osteoporosis diagnostic system. Second, this retrospective study, conducted at a single institution, had variations in the intervals between bilateral THA surgeries and included a limited number of cases. The errors of these AI systems and the small sample size are significant limitations that may affect the robustness of this study. Further research is needed to improve the accuracy of the AI systems themselves and to increase the sample size through multi-center studies. Moreover, although this study stratified patients by age and excluded those who had undergone osteoporosis treatment, it is also a limitation that factors such as activity level, including the patients' exercise habits, and dietary intake were not considered. Future studies that take these patient backgrounds into consideration may have the potential to address this limitation. Additionally, we compared the BMD of THA patients with age-matched reference values from previous reports of the general population, and a power analysis was not conducted. In the future, analyzing and comparing the natural course of BMD in hip OA cases that did not undergo surgery will allow for a study more focused on the effects of THA.

Despite these various limitations, the strength of this study lies in its innovative approach as the first to use an AI-assisted osteoporosis diagnostic system to comprehensively track changes in BMD before and after THA, providing a foundation for future research.

## Conclusions

THA has the potential to attenuate age-related BMD decline in postmenopausal women with hip OA. However, this finding should be interpreted with caution, as the effect may be influenced by previously mentioned limitations, and further research is needed to determine its applicability to the broader postmenopausal population.
